# A new long term gridded daily precipitation dataset at high-resolution for Cuba (CubaPrec1)

**DOI:** 10.1016/j.dib.2023.109294

**Published:** 2023-06-03

**Authors:** Abel Centella-Artola, Arnoldo Bezanilla-Morlot, Roberto Serrano-Notivoli, Ranses Vázquez-Montenegro, Maibys Sierra-Lorenzo, Dayron Chang-Dominguez

**Affiliations:** a Institute of Meteorology, Loma de Casa Blanca, Regla, La Habana 11700, Cuba; b Department of Geography and Regional Planning, Environmental Sciences Institute (IUCA), University of Zaragoza, Zaragoza 50009, Spain; c University Otto-von-Guericke of Magdeburg, Universitätsplatz 2, Magdeburg 39106, Germany

**Keywords:** Rainfall quality control, Rainfall time series reconstruction, Rainfall spatial distribution

## Abstract

The paper presents a high-resolution (-3km) gridded dataset for daily precipitation across Cuba for 1961-2008, called CubaPrec1. The dataset was built using the information from the data series of 630 stations from the network operated by the National Institute of Water Resources. The original station data series were quality controlled using a spatial coherence process of the data, and the missing values were estimated on each day and location independently. Using the filled data series, a grid of 3 × 3 km spatial resolution was constructed by estimating daily precipitation and their corresponding uncertainties at each grid box. This new product represents a precise spatiotemporal distribution of precipitation in Cuba and provides a useful baseline for future studies in hydrology, climatology, and meteorology. The data collection described is available on zenodo: https://doi.org/10.5281/zenodo.7847844

**Specifications Table**

**Table:** 

Subject	Earth and Planetary Sciences
Specific subject area	Climatology, Meteorology, Hydrology
Type of data	Georeferenced gridded daily precipitation values
How the data were acquired	The daily data series were provided by the National Institute for Water Resources of Cuba (NIWR) upon request of the Institute of Meteorology of Cuba. These data comprises a subset of 630 stations which fully covers the period from 1961 to 2008 with a very low amount of missing information. No objective quality control process is applied to this raw information.
Data format	Analyzed
Description of data collection	The dataset is a gridded precipitation product of daily precipitation for the period 1961-2008 at 0.027˚ spatial resolution.
Data source location	Institution: Institute of Meteorology of Cuba Country: Republic of Cuba Longitude and latitude for collected data: 84.95˚W – 74.16˚W, 19.84˚N - 23.14˚N
Data accessibility	Repository name: Zenodo Direct URL to data: https://doi.org/10.5281/zenodo.7847844

**Value of the Data**

• This article describes the development of a long-term (48 years) gridded daily precipitation dataset at high spatial resolution (−3 km). This is the first rainfall product for Cuba built with information from more than 600 rain gauges, after an objective quality control process.
• The dataset provides usable and valuable information for climate variability studies as well as for the assessment of precipitation extremes. Also, it could be an important reference for numerical weather and climate model evaluation and for empirical sub-seasonal and seasonal model development with high spatial resolution.
• This gridded precipitation product also includes the estimation error for every grid box and time step. This piece of full inside information provides an idea about the estimation uncertainty and is the benchmark useful when comparing with similar products.

## 1 Objective

**Fig. 1:**
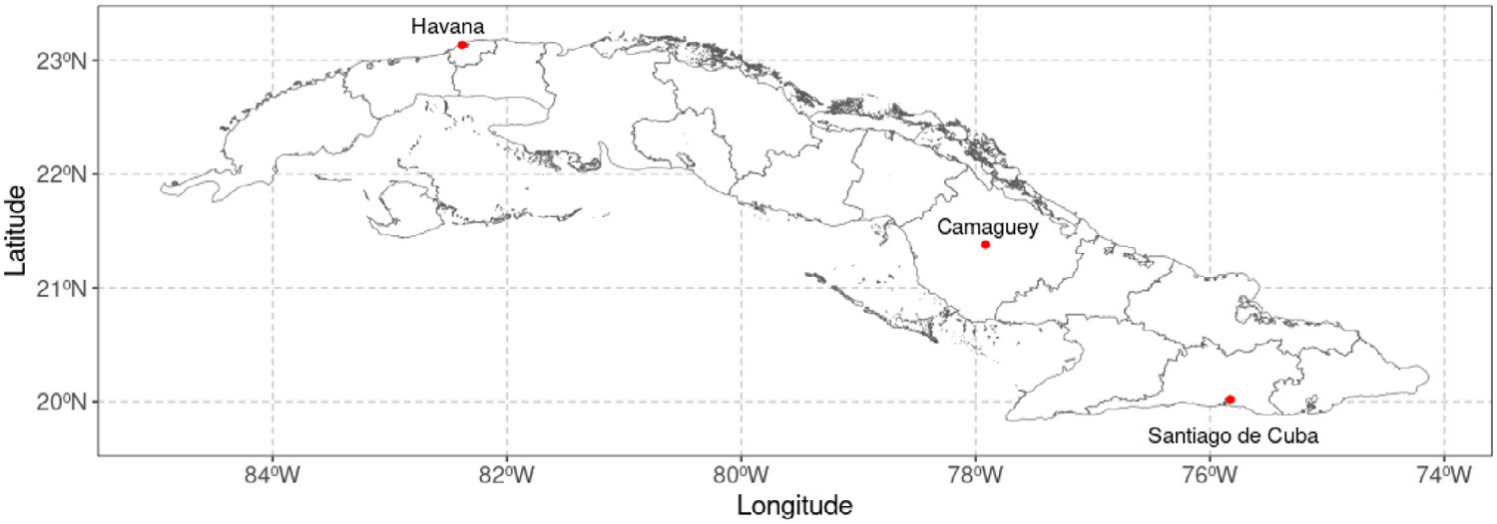
Map of the republic of Cuba. Red points indicates the three biggest cities in Cuba.

Precipitation is a meteorological variable that has a great influence on human activities and it is also considered one of the most important variables in the dynamic of the atmosphere. In the previous context long-term gridded datasets based on accurate observational data are relevant for research related to climate variability, climate change, and hydrological modeling, among others. The dataset presented in this article has been developed to be considered as reference data for a research project aimed at developing seasonal prediction of rainfall and drought for the Cuban territory (Fig. 1). Furthermore, research such as developing climate change scenarios and the study of observed climate variations in Cuba may also benefit from the information from this dataset. The dataset is available in NetCDF format to enable its use with a wide range of open-source softwares.

## 2 Data Description

The dataset comprises five files of daily precipitation estimates (millimeters) covering Cuba with a spatial resolution of 0.027˚ × 0.027˚ from 1961 to 2008. In addition to daily precipitation magnitudes, the estimation error is also included, which offers a measure of the uncertainty for each rainfall estimate. The files are provided in NetCDF format, a self-describing file format that can be read using a wide range of scripting languages such as R, Python, CDO, GrADS, Matlab, among others. The files are made up of three dimensions (longitude, latitude, and time), as well as two variables named pr and pr_er, which are precipitation and estimation error, respectively.

**Fig. 2:**
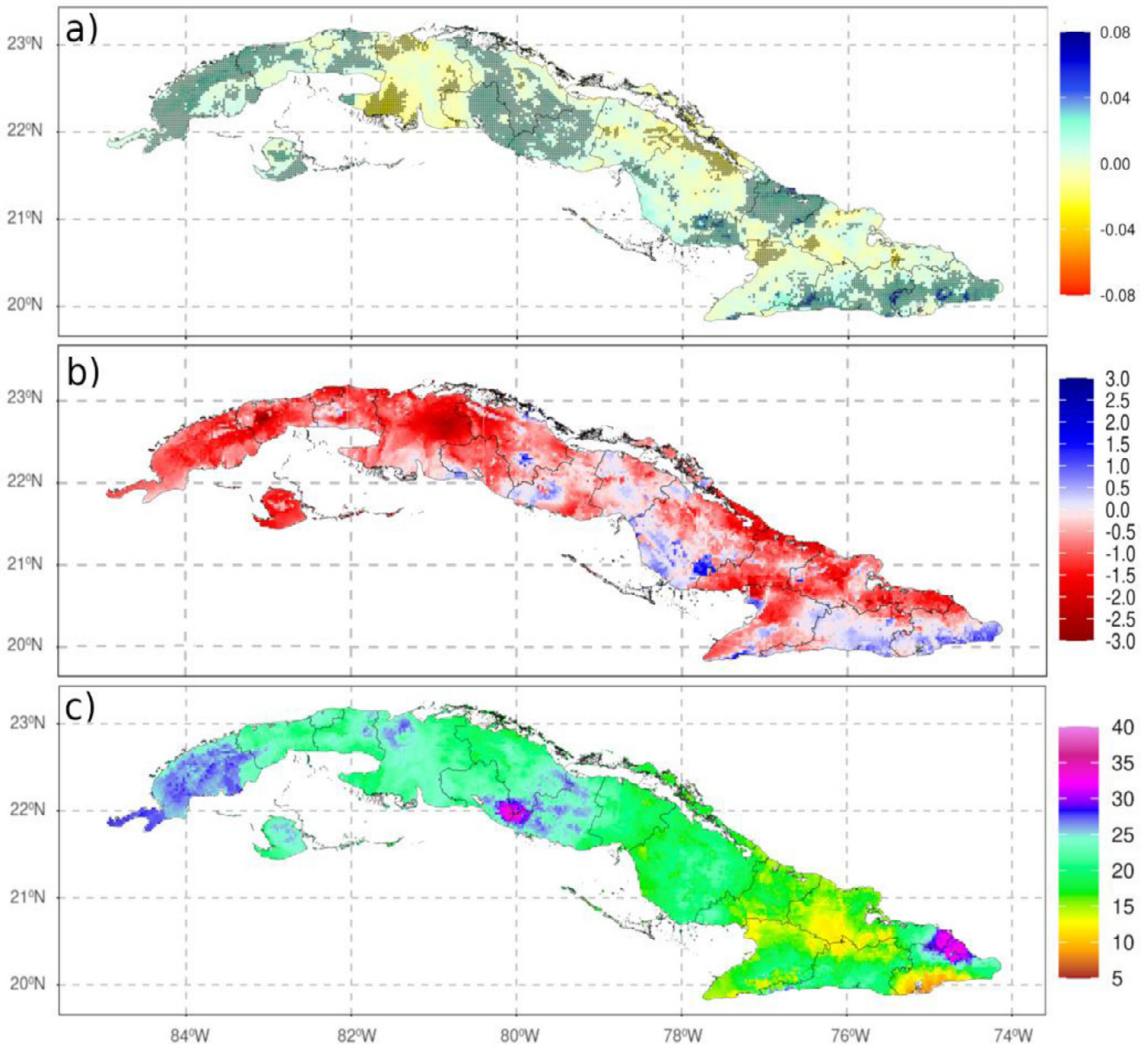
(a): Spatial distribution of the SPI12 for September 1986; b): SDII trend (mm/10-years) for 1961-2008 period, dotted areas indicate statistically significant trends at 95% of confidence; c): Spatial distribution of the uncertainty (mm).

The dataset offers the possibility of performing a wide range of climatic analyses of precipitation variability throughout Cuba, such as average rainfall amounts, spatially differenced trends, characterization of extreme events, etc. As an exemplar of its versatility, Fig. 2a shows the spatial distribution of the Standardized Precipitation Index for 12-month scale (SPI12) [1] for September 1986, where spatial differences in drought severity can be related to different geographical constrains. Fig. 2b shows the trend of rainfall intensity in the period 1961-2008, which is determined by the Simple Daily Intensity Index (SDII). Acording to Klein et al. [2] SDII was calculated as the annual mean precipitation on wet days (days with precipitation greater than 1 mm). As single time series are encoded within each grid box, calculations at each temporal resolution such as trends and their significance are straightforward. Lastly, Fig. 2c shows the spatial distribution of the mean estimation errors (measured in mm), which offers an idea of the areas about the uncertainty in the estimates. A measure of error provides a useful indicator robustness of climatic analyses but also an added value since the areas with higher uncertainty represent zones where precipitation differs more than others and, as a consequence, it is more difficult to provide reliable estimates or forecasts.

## 3 Experimental Design, Materials and Methods

### 3.1 Overview

**Fig. 3:**
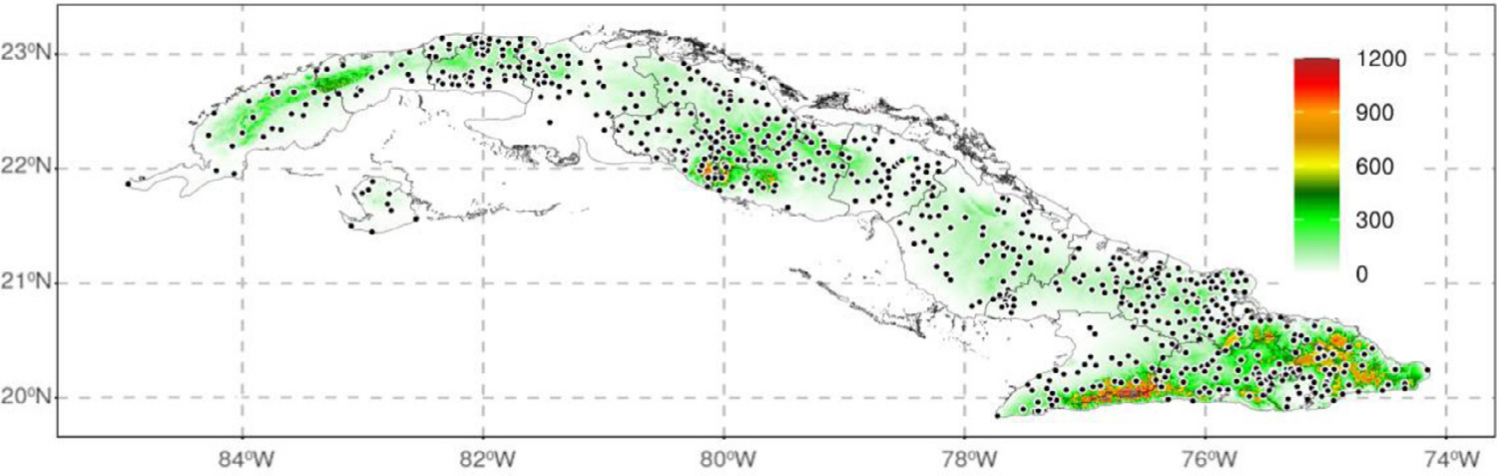
Location of the 630 rain-gauges whose time series were used to create the gridded dataset (black dots). The shaded color area represents elevation in meters above sea level.

The initial data was provided by the National Institute of Water Resources of Cuba (NIWR) (https://www.hidro.gob.cu/es). It consisted of the time series from 630 rain gauges, sourcing from the network of stations operated by NIWR . The NIWR maintains this database, considering that it covers a long period (1961–2008), with a minimum of missing information and with a relatively good and even spatial distribution in almost all regions of Cuba (Fig. 3). It should be highlighted that the data after 2008 were not available with enough quality and completeness to be considered in developing the gridded dataset. A future version of this gridded product will include the recent period once NIWR ends the process to complete the amount of information with satisfactory quality and a reduced number of missing data.

The gridded dataset presented here was built from these original 630 stations by creating reference values (RVs) using Generalized Linear Models (GLM) based on the rainfall values of the 10 nearest observations and its altitude, longitude, and latitude as covariates. All the calculations were developed in three main stages using the R package reddPrec (https://cran.r-project.org/web/packages/reddPrec), which contains specific functions for: 1) quality control, 2) reconstruction of original daily precipitation series and 3) estimation of the rainfall values over a regular grid [3].

### 3.2 Quality Control Process

The quality control (QC) scheme is based on the comparison of the values registered in each location, with their corresponding reference values (RV), that are calculated with the information of the 10 closest stations (NNS). The RV is a prediction of 1) the probability of precipitation occurrence and 2) the magnitude of daily precipitation (if probability is significant). It is computed from a GLM where the observations act as dependent variables, and the associated geographical information of each station participating in the model (latitude, longitude, altitude) is the independent variable. The RV then is determined from two estimates: i) a binomial prediction (BP) of the probability of occurrence of a wet day; and ii) an estimate of the magnitude (MP) of precipitation. Thus, RV is equal to MP if BP is greater than 0.5 (probability higher than 50%) and equal to zero otherwise. More details on Serrano-Notivoli et al. [3].

This first set of RV is used to develop the quality control process using the *qcPrec* function from reddPrec package in order to detect suspicious data by comparison with the original information. This function performs a sequential process that includes the checking of the following criteria: 1) Suspect individual rain (QC1): the observed value is greater than zero and all 10 NNS are zero; 2) Suspect individual zero (QC2): The observed value is zero and all of its 10 NNS are above zero; 3) Suspect outlier (QC3): The magnitude of the observed value is 10 times greater or less than that predicted by its 10 NNS; 4) Suspect dry (QC4): the observed value is zero, the probability of a wet day is greater than 0.99, and the estimated magnitude is greater than 5 mm; and 5) Suspect wet (QC5): the observed value is greater than 5 mm, the probability of dryness is greater than 0.99, and the estimated magnitude is less than 0.1 mm. The QC process is iterative using the 10 NNS without suspect values and stop when no more observations are flagged.

**Fig. 4:**
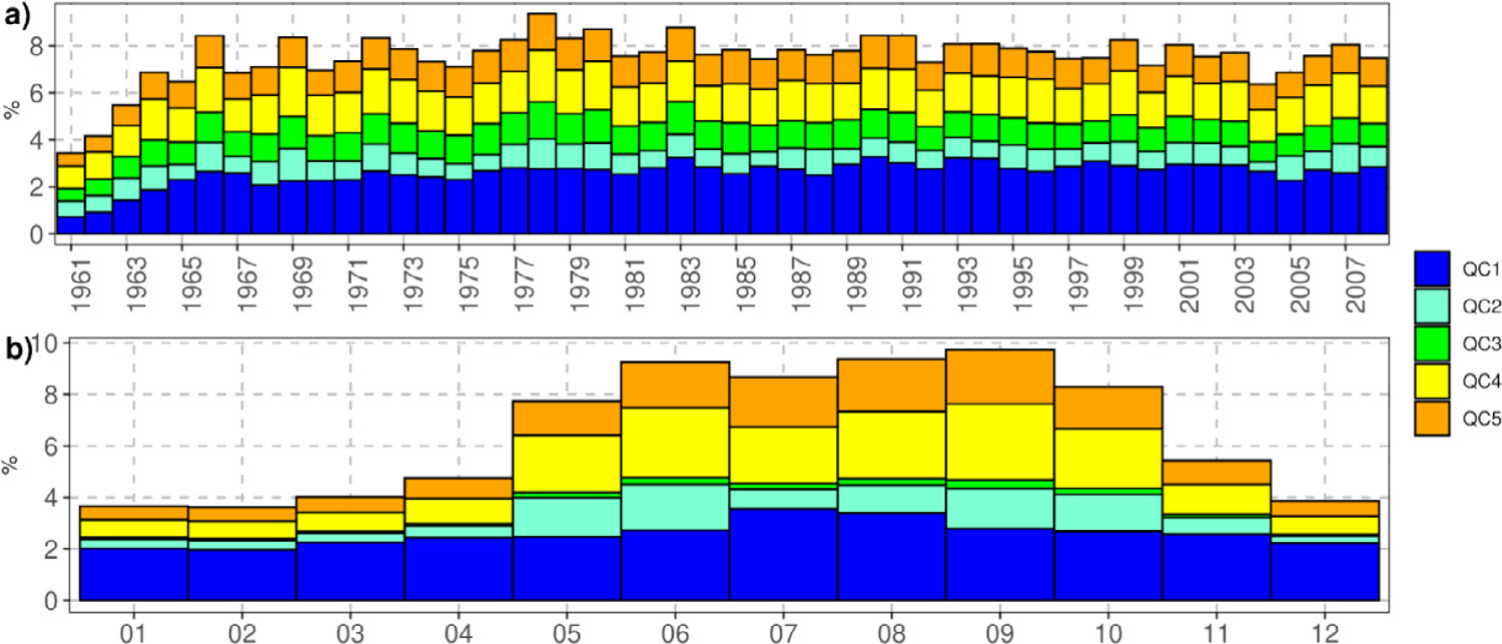
Frequency of suspect data by criteria. (a) per years and (b) per months

In general, the number of suspected cases in the analyzed period is relatively low, with around 6-9% of the observations per year (Fig. 4a), although in the first three years were lower that 6%. As a rule, the greatest contribution is made by the suspect data from QC1 and QC4 (2.6 and 1.7%, respectively). Commonly, these are the most frequent types when using this quality control [3]. For QC1, the detection rate of suspects is relatively stable across the year (Fig. 4b), but the rate for QC4 is high during the rainy season.

### 3.3 Gap Filling Process and Time Series Reconstruction

After the QC process, all suspect values were removed from the original data series. Then, these clean series were used to calculate a new set of RV. Since the RV are always calculated for all days and locations without the influence of the recorded value at the target station, the comparison between the RV and the corresponding observed (OBS) values is considered as a leave-one-out cross-validation (LOO-CV). We then use these pairs of values to compute three metrics of goodness-of-fit statistics to assess the agreement between RV and OBS: mean error (ME), mean absolute error (MAE), and the modified efficiency index of Kling-Gupta (KGE) [4]. In general, the ability of the reconstruction method to estimate daily precipitation at different locations is appropriate with ME, MAE and KGE values of -0.02, 2.01 and 0.79, respectively.

We also assessed the ability of the methodology to correctly estimate the occurrence of zero (dry) and non-zero (wet) values, as well as its accuracy in predicting the precipitation magnitude. In the first case, results showed that the method is not biased in the estimation of dry and wet days considering that the number of zeros observed is 8,334,787 and the estimate is 8,448,217 (a ratio of 0.9865). In all cases the rate or precision of true zero (OBS = 0 and RV = 0) as well as true positive (OBS > 0 and RV > 0) were over 92 and 70% respectively, whereas the rates of false zero (OBS > 0 and RV = 0) and false positive (OBS = 0 and RV > 0) were notably lower (7.6% and 29.0%, respectively). Overall, the false zeros (FZ) and false positives (FP) were largely related to prediction complications on days with very low rainfall values. For example, in 36.4% of the FP the estimated values were less than 1 mm. While this difference is small in magnitude, it becomes apparent in the dry or wet day precision. To a large extent, these results are consistent with those reported by other studies [5,6].

**Fig. 5:**
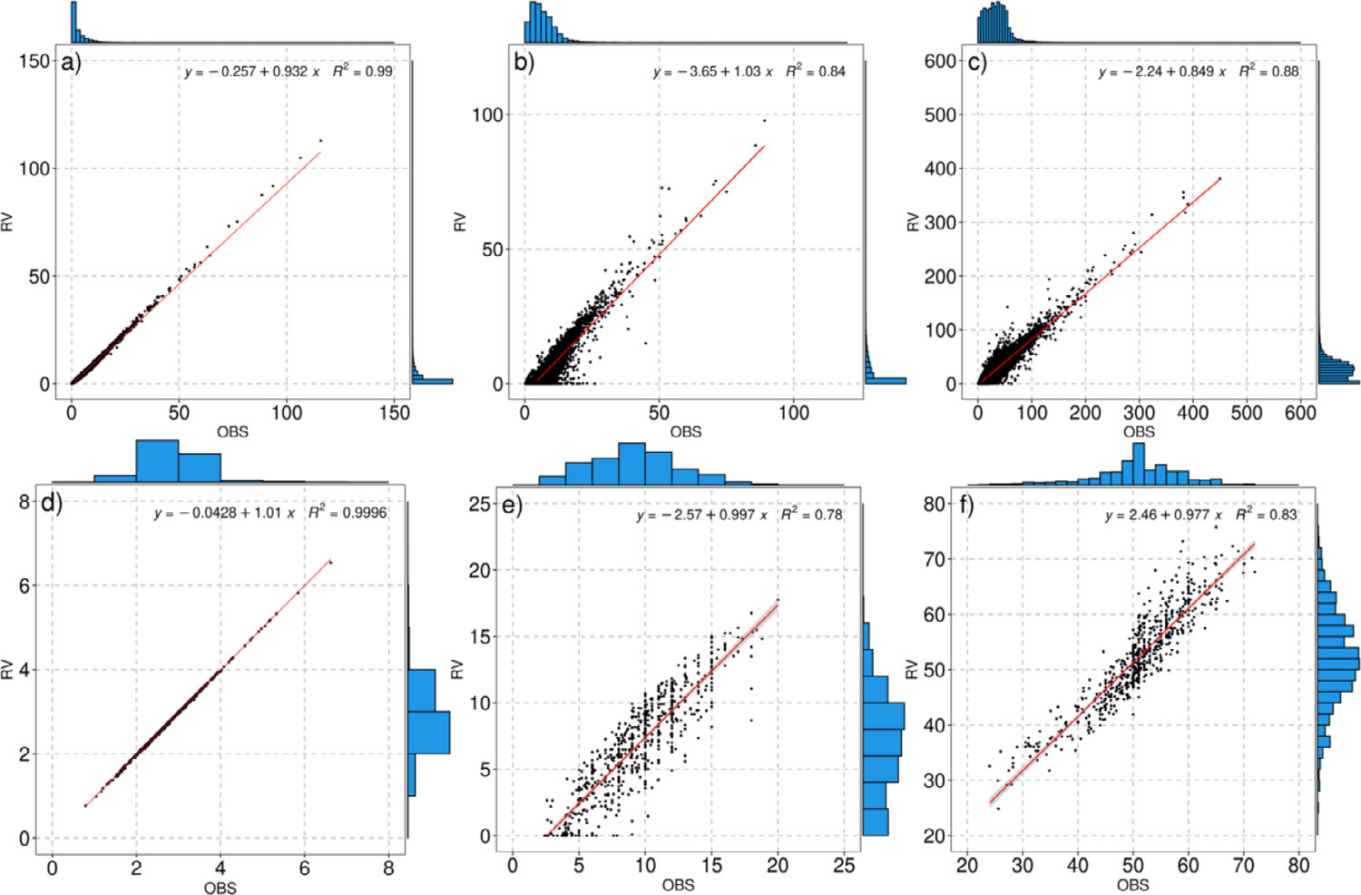
Comparison between estimates and observations by days (a,b,c) and stations (d,e,f). (a and d) compare mean precipitation; (b and e) the median of the wet days and; (c and f) the 95^th^ percentile in wet days. Each panel includes the regression line and the value of the Pearson correlation coefficient. The marginal plots are the histograms of the observed and estimated values.

The evaluation of the prediction of the magnitudes of rainfall was performed by: i) comparing the observed and estimated values by days (average of all the stations for each day) and ii) by stations (average of all the values for each station). In both cases, the comparison considers the mean precipitation, the median number of wet days, and the median number of days with rain above the 95th percentile. As can be seen in Fig. 5, the highest Pearson correlation coefficient values were reached for both the daily and stations means (Fig. 5a,d). These high scores are influenced by the predominance of zero values in the observed data which, as seen before, are estimated with considerable precision. However, for wet days and extremes, the agreement is weaker and the correlation scores decrease, although still higher than 0.75 (0.84 and 0.76 for days, and 0.78 and 0.83 for seasons). In part this reduction is due to the more frequent prediction of low values and possibly the higher frequency of false positives mentioned above. According to Fig. 5(c and f), the effect of the prediction of low values becomes more evident in the comparison by days over by stations, while a slight overestimation is observed in several stations with extreme rainfall greater than 50 mm (Fig. 5f). In general, there is a high predictive ability.

### 3.4 Gridding Process

To produce the final dataset, we used the reconstructed daily time series and applied the same procedure based on the calculation of RV over a 3 × 3 km spatial resolution grid, which is a good compromise between the spatial extent and the station network density [7]. Thus, for each grid box (longitude, latitude, and altitude) and each day of the total period, RV were computed based on the data of the 10 closest reconstructed stations. We also computed the standard error (in mm) for each RV, which can be used as a measure of uncertainty. The ratio between this error and the RV results in a relative error (expressed in percentage) in each aggregation (monthly, annual, and seasonal).

## Ethics Statements

This dataset does not include any human subjects, animal experiment, or social media platforms.

## CRediT authorship contribution statement

**Abel Centella-Artola:** Conceptualization, Methodology, Formal analysis, Data curation, Writing – original draft. **Arnoldo Bezanilla-Morlot:** Methodology, Data curation, Writing – original draft. **Roberto Serrano-Notivoli:** Validation, Software, Writing – review & editing. **Ranses Vázquez-Montenegro:** Data curation, Writing – review & editing. **Maibys Sierra-Lorenzo:** Data curation, Writing – review & editing. **Dayron Chang-Dominguez:** Writing – review & editing.

## Declaration of Competing Interest

The authors declare that they have no known competing financial interests or personal relationships that could have appeared to influence the work reported in this paper.

## Data Availability

CubaPrec1: A 48 years long term gridded daily precipitation dataset at very high-resolution for Cuba. (Original data) (zenodo).
